# Equine Respiratory Medicine and Cardiology

**DOI:** 10.3390/ani13182952

**Published:** 2023-09-18

**Authors:** Luca Stucchi, Chiara Maria Lo Feudo, Francesco Ferrucci

**Affiliations:** 1 Department of Veterinary Medicine, University of Sassari, Via Vienna 2, 07100 Sassari, Italy; lstucchi@uniss.it; 2 Department of Veterinary Medicine and Animal Sciences, University of Milan, Via Università 6, 26900 Lodi, Italy; francesco.ferrucci@unimi.it

In the last few years, the attention regarding the health of the lungs and heart of equine patients has been continuously growing. Indeed, if we search for studies categorized in the PubMed database on the topic “equine respiratory medicine”, we can find that 980 articles have been published in the last decade [1]. Interestingly, although the first description of heaves as an “asthma-like” condition dates back to the 1960s [2], the recent revision of the nomenclature for chronic inflammatory diseases affecting the equine lower airways [3] brought about a rapid expansion of the knowledge about these conditions. Similarly, the progressive increase in the life expectancy of horses led to a rise in the diagnosis of cardiac problems. Consequently, the rapid advance of research in the cardiology field led to the availability of new technologic tools, such as three-dimensional electro-anatomical mapping of the equine heart [4]. These tools allow innovative diagnostic and therapeutic approaches that, until a few years ago, were considered impossible. Finally, the growing awareness and sensitivity of owners and trainers about the equine welfare played a fundamental role in this positive and rapid evolution.

The content of this Special Issue aims to reflect the current direction of research, dealing with a wider understanding of the pathophysiological mechanisms underlying respiratory and cardiac diseases of horses, as well as the development of novel diagnostic and therapeutic approaches that are essential to improve their management.

For instance, the review by Kozlowska et al. [5] highlights the relevance of new imaging techniques, such as computer tomography and magnetic resonance, in the diagnosis of upper respiratory tract diseases. Moreover, the authors explore the most recent advances in lung function testing techniques, describing the usefulness of electrical impedance tomography and the impulse oscillometry system (IOS). On this last topic, the original article by Stucchi et al. [6] reports the data of IOS measurements in severely asthmatic horses in different stages of the disease, describing for the first time the phenomenon of the expiratory airflow limitation during the exacerbation phase. Mainguy-Seers et al. [7] performed IOS measurements on asthmatic mares in their study, identifying a positive effect of the luteal phase of the estrus cycle on lung function parameters and hypothesizing a role of sex hormones in equine asthma pathophysiology.

Another key theme in the current literature is represented by the complex immunological pathways involved in equine asthma (EA). The review by Simões et al. [8] widely explores the different phenotypes and endotypes of EA, and the current knowledge concerning the cytokines, the inflammatory biomarkers, and the microbiome involved in the development of the disease. The review by Klier et al. [9] proposes a novel immunomodulatory treatment for EA, based on the inhalation of nanoparticulate cytosine–phosphate–guanine oligodeoxynucleotides, which may also find application in asthmatic human patients. Finally, the paper by Basano et al. [10] reports for the first time a positive association between the presence of giant multinucleated cells and mast cells in the broncho-alveolar lavage fluid of EA-affected horses.

This Special Issue also investigates the different respiratory diseases commonly observed in racehorses, such as dynamic upper airway obstructions (DUAOs), exercise-induced pulmonary hemorrhage (EIPH), and mild–moderate equine asthma (MEA). In the first of their two retrospective studies, Lo Feudo et al. [11] identified no association between the presence of DUAOs and lower airway inflammation in Thoroughbred and Standardbred horses, suggesting that disorders of the upper and lower airways follow independent pathological pathways. Moreover, while they did not detect a role of the size of the epiglottis in the development of DUAOs, they reported an association between its flaccid appearance observed during resting endoscopy and the development of dorsal displacement of the soft palate during exercise. In the second article by Lo Feudo et al. [12], the effects of EIPH on fitness parameters, measured through a standardized treadmill test, of Standardbred trotters were investigated. The study concluded that EIPH does not affect the athletic capacity of racehorses, and its role in decreased performance quality may follow a different pathway. Furthermore, the prospective case–control study by Stucchi et al. [13] evaluates the efficacy of the administration of a nutraceutical supplement, composed of different herbal extracts with antioxidant properties, in Thoroughbred racehorses affected by MEA. The results show a reduction in tracheal mucus accumulation and clinical score in the treated patients.

Lastly, the focus of the paper of Marzok et al. [14] is a species that has gained more and more popularity as a companion animal in recent years: the donkey. The authors studied the reference values and repeatability of pulsed-wave Doppler echocardiographic variables, improving the limited availability of data reported in the literature for these animals.

In conclusion, we believe that the papers published in this Special Issue could enrich the current knowledge of equine cardio-respiratory medicine with innovative, interesting, and useful data, and we hope that readers will be positively impressed.
